# Content of Selected Harmful Metals (Zn, Pb, Cd) and Polycyclic Aromatic Hydrocarbons (PAHs) in Honeys from Apiaries Located in Urbanized Areas

**DOI:** 10.3390/foods13213451

**Published:** 2024-10-29

**Authors:** Aleksandra Wilczyńska, Natalia Żak, Ewa Stasiuk

**Affiliations:** Department of Quality Management, Gdynia Maritime University, ul. Morska 81-87, 81-225 Gdynia, Poland; n.zak@wznj.umg.edu.pl (N.Ż.); e.stasiuk@wznj.umg.edu.pl (E.S.)

**Keywords:** environmental pollution, honey, polycyclic aromatic hydrocarbons, harmful metals

## Abstract

The chemical composition of honey, and therefore its quality and properties, is influenced by many factors, including its botanical origin and the harvesting conditions—the location of the apiary, access to melliferous plants, the proximity of industrial infrastructure and communication routes, etc. This quality may be reduced by undesirable, toxic compounds that penetrate honey from a contaminated environment, such as heavy metals and residues from other environmental pollutants. Therefore, the aim of our research was to assess the quality of honeys from urbanized areas—in particular, to assess contamination with heavy metals and persistent organic pollutants (PAHs). In total, 35 samples from six different apiaries located in urbanized areas were examined. The content of heavy metals (Cd, Pb, Zn) was determined by atomic absorption spectrophotometry (AAS), and the content of total PAHs as the sum of the concentrations of the compounds benzo(a)anthracene, chrysene, benzo(b)fluoranthene and benzo(a)pyrene was determined by high-performance liquid chromatography with fluorescence detection (HPLC-FLD). The average zinc content ranged from about 2 to 4.5 mg/kg, the average lead content ranged from 3.5 µg/kg to 388 µg/kg and the average cadmium content ranged from 0.5 to 14 µg/kg. It was found that all honeys contained certain amounts of harmful metals, and only lead exceeded the permissible limits. None of the samples tested contained sum content of PAHs exceeding 10 µg/kg of honey. Contrary to our expectations, the results obtained indicate that honeys from urbanized areas do not contain these harmful substances. In general, the presence of harmful metals does not, however, reduce honey’s quality or its health value.

## 1. Introduction

Honey is one of the most valuable components of the human diet, and its properties have been known and exploited for centuries. Honey is a source of energy; has bactericidal, anti-inflammatory, antioxidant and anti-cancer effects; is used to treat wounds and ulcers; relieves stress; and supports the treatment of diseases of the cardiovascular, digestive and respiratory systems and kidney function, as well as convalescence [1,2,3,4,5]. The therapeutic and prophylactic effects of honey are closely related to its chemical composition, which is influenced by many factors, including its botanical origin, the harvesting conditions (location of the apiary, access to melliferous plants, proximity of industrial infrastructure, communication routes, etc.), the storage conditions and the conditions of consumption [6,7]. According to literature sources, honey contains over 300 substances, classified into various groups of chemical compounds and characterized by different types of biological activity [2,8,9,10,11,12]. However, honeys may also contain undesirable compounds that penetrate from a polluted environment or as a result of the improper use of chemotherapeutics to combat bee diseases—acaricides and antibiotics. Compared to other bee products, honey contains relatively few contaminants. The most common contaminants in honey include acaricides, heavy metals and pesticides. Honey has been found to contain heavy metals such as mercury (Hg), zinc (Zn), lead (Pb), arsenic (As), copper (Cu) and cadmium (Cd). Among them, the most harmful are mercury, cadmium, zinc and lead [13,14,15,16,17]. All of the abovementioned elements are widely distributed in all elements of the environment; therefore, they may have a harmful effect on both plant and animal organisms. Cadmium inhibits the germination of seeds, plant growth and nutrient distribution, as well as photosynthesis. It disrupts the activity of many enzymes, which, in animal organisms, can lead to iron deficiency, anemia, kidney and cardiovascular diseases, hypertension, atherosclerosis, cancer and osteoporosis. According to the International Agency for Research on Cancer (IARC), cadmium is classified as group 2B. It is most likely responsible for lung and prostate cancer [18]. Lead has a similar effect: it inhibits the activity of many enzymes, which results in impaired oxygen transport, impaired energy metabolism, weakened defense mechanisms against free radicals, disrupted intra-organ information transfer, anemia and disorders in the peripheral nervous system—lead encephalopathy and kidney and liver disorders [19]. Zinc plays an important role as an essential trace element in all living systems, from bacteria to humans. The toxicity of zinc and most zinc-containing compounds is generally low. However, manifestations of overt toxicity symptoms (nausea, vomiting, epigastric pain, lethargy and fatigue) will occur with extremely high zinc intake [20]. 

The available literature indicates great variation in the content of individual harmful metals in honey. Bees, in search of food, undertake long flights. The area visited by bees is closed in a circle with a radius of 2 km, and, in certain conditions, their flight range can be as large as 7 km. As a result, the hive receives material from a large area. Harmful elements can come from both the soil and the air. Low-mobility metals, including lead, come mainly from polluted air; lead contamination is of a surface nature. Other metals, e.g., zinc and cadmium, which are characterized by high mobility, especially in conditions of a reduced soil pH, enter the nectar from the soil via the trophic chain, through the root system, stems and flowers [13].

Other environmental pollutants that may negatively affect the quality of honey are pesticide residues and persistent organic pollutants, including PAHs [21,22,23,24]. Polycyclic aromatic hydrocarbons (PAHs) consist exclusively of carbon and hydrogen. They are connected by benzoic ring systems (from simple to complex) that give a wide spectrum of various physical, chemical and toxicological properties [25,26,27]. We distinguish two groups of hydrocarbons: light, so-called LMW (containing up to four rings), which decompose faster, and heavy, so-called HMW (containing more than four rings), which decompose more slowly and are carcinogenic [28,29,30]. It is possible to classify about 200 PAHs that exhibit toxic effects. One of the PAHs considered when determining air quality is benzo(a)pyrene [28]. PAHs are complex hazardous organic compounds that are released into the atmosphere as by-products of partial combustion processes. They are widespread environmental pollutants that have harmful biological, toxic, mutagenic and carcinogenic effects [25]. Their presence in the ecosystem is one of the greatest problems of the modern world, and their toxicity affects not only humans but also plants, microorganisms and other living organisms [29].

The origins of PAHs can be both natural (e.g., forest fires, volcanic eruptions) and secondary, e.g., as a result of human activity or with its participation (industrial processes, vehicle emissions, waste incineration, food processing, etc.) [31,32,33,34,35,36,37,38,39,40,41,42,43,44,45].

There are numerous studies conducted worldwide on the level of PAHs in the natural environment, primarily air, soil and sediments, as well as surface water, groundwater and water runoff from roads [46,47,48]. For example, PAH concentrations ranging from 1 mg/kg to over 300 g/kg have been recorded in soil [25,29,48]. Polycyclic aromatic hydrocarbons belong to the group of persistent organic pollutants, which means that these toxic compounds can accumulate in the soil and remain in the environment for a long time [29,30,49]. Then, they are transferred from the atmosphere to vegetation, medicinal herbs and food [49,50,51]. It can be said that their process of movement in the natural environment is closed. The final recipients are humans, for whom PAHs are highly toxic [23]. Polycyclic aromatic hydrocarbons (PAHs) are substances that are also present in food. Their presence has been recorded in roasted, grilled, fried and smoked products that have undergone thermal processing. The risk associated with the presence of PAHs in food is quite high, because they have a harmful effect on the human body—toxic, mutagenic and carcinogenic effects [52,53,54]. According to information provided by the World Health Organization (WHO), exposure to PAH substances is, in 99% of cases, caused by food (absorption through the oral route), and, in less than 1%, they enter the human body through the respiratory system (inhalation). Scientific studies prove that PAHs occur in food. For example, Veiga et al., in 2014, indicated the presence of PAHs in roasted Guarana seeds [55]. The presence of these substances was also noted in rice [56], grilled and smoked meat [57], roasted coffee [58] and honey [52,59]. The European Commission’s Regulation No. 915/2023 specifies the maximum levels of PAHs in various food products—for honey, this level is 0.10 mg/kg [52,60].

The most commonly used analytical methods for the determination of carcinogenic PAHs in food products are high-performance liquid chromatography (HPLC) with a selective fluorescence detector (FLD) and high-resolution gas chromatography coupled with mass spectrometry (GC-MS). The GC-MS method is usually recommended when the chemical compound cannot be easily identified by HPLC. Dennis et al. compared the above methods, taking into account such parameters as the recovery, separation quality, analysis time and cost of execution. They showed that the repeatability of both methods was very good, within 10%. Both methods performed well, allowing for the comparison of data with a wide range of values (0.2–1000 μg/kg). Capillary GC had much higher resolving power, so a larger number of PAHs could be determined and separated by this method, but HPLC was able to separate individual isomers, so it had higher selectivity [61].

As the source of the above substances in food products is mainly the polluted environment, the aim of our research was to assess the quality of honeys from urbanized areas—in particular, to assess contamination with toxic metals (Zn, Cd, Pb) and PAHs. The concentrations of these pollutants were examined mainly because of their toxic and carcinogenic effects. The subject of our research was honey from apiaries located in large Polish metropolises. In our country, the first urban apiaries appeared in Warsaw; currently, in Warsaw alone, there are already 400 city hives. These apiaries serve to draw attention to the need for the presence of honeybees in the environment and to the problems of pollinating insects, but various products are also obtained from them, such as honey, pollen, propolis, etc. Scientific research on urban apiaries focuses primarily on assessing the content of heavy metals in the bodies of bees, honey, pollen and propolis. However, no one in Poland has yet comprehensively determined the content of both heavy metals and other persistent environmental pollutants in urban honeys.

## 2. Materials and Methods

### 2.1. Sample Collection

**Figure 1:**
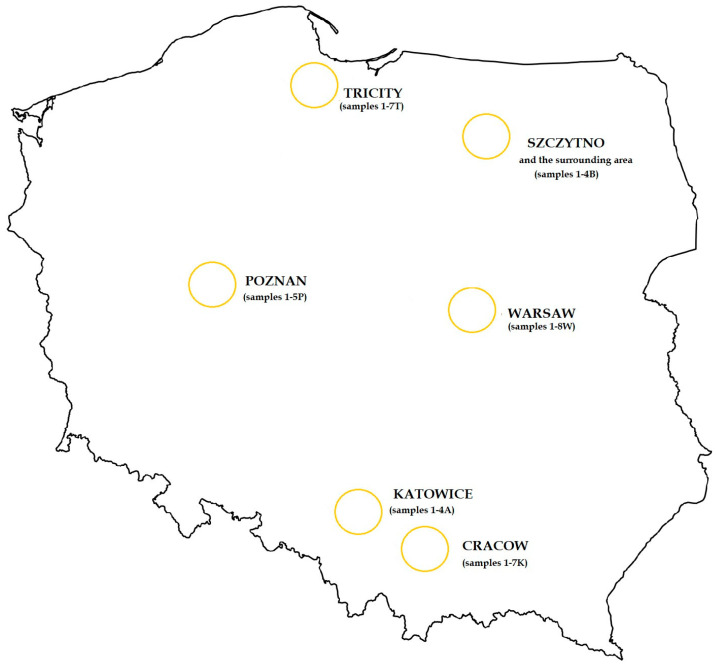
Origin of the tested honey samples.

This study involved the analysis of a total of 35 honey samples from the 2024 harvest season, collected in 6 different regions of Poland: 7 in the Tri-City (North Poland, T), 8 in Warsaw (Central Poland, W), 5 in Poznań (West Poland, P), 7 in Cracow (South Poland, K), 4 in Katowice (South Poland, A) and 4 in Szczytno (Northeastern Poland, B) (Figure 1). The honeys were obtained directly from apiaries located in city centers or near roads with heavy traffic.

### 2.2. Heavy Metal Determination

The content of heavy metals (Cd, Pb, Zn) was determined by atomic absorption spectrophotometry on a Varian AA (SpectrAA-20 Plus, Varian, Belrose, Australia), after the prior mineralization of the sample [62]. For mineralization, about 1.3 g of honey was weighed with accuracy of 0.001 g directly into an XP-1500 vessel (CEM Corporation, Matthews, NC, Canada) Then, 4 mL of redistilled water and 2.5 mL of nitric acid (Suprapur Nitric Acid 65%, Supelco, Germany) were added. The vessels’s content was mineralized in a MARS 5X microwave oven (CEM Corporation, Matthews, NC, Canada) at a temperature of 240 °C and pressure of 150 psi (~1 MPa). After mineralization, the crucible’s content was quantitatively transferred to a 50 mL volumetric flask and topped up with 0.1% HNO_3_. After this, the concentration of heavy metals was determined using a Varian AA atomic absorption spectrophotometer with a graphite furnace atomizer and an acetylene–air flame; for lead (Pb) and cadmium (Cd), the Palladium matrix modifier (Sigma-Aldrich, Germany) was also used in the measurements. The measurement wavelengths for different heavy metals were as follows: cadmium (Cd) (228.8 nm), lead (Pb) (283.3 nm) and zinc (Zn) (213.9 nm). A slit width of 0.5 nm was used for lead (Pb) and cadmium (Cd), while a slit width of 1.0 nm was used for zinc (Zn). The content of individual elements was evaluated based on the standard curves; standard solutions were prepared using serial dilutions of 1000 mg/L single-element standards in 2% HNO_3_ (Cadmium Standard for AAS, Supelco, Germany, Lead Standard for AAS, Supelco, Germany, Zinc Standard for AAS, VHR Chemicals, Thermo Scientific, Waltham, MA, USA). First, absorption in standard solutions of individual metals was measured; then, absorption measurements were taken in mineralized samples. The absorption measurement for the standard solutions was repeated every 20 subsequent samples. In order to check the analytical procedure, internal quality control was used, consisting of testing the same sample of a known concentration at different times. The limits of detection and quantification (LOD and LOQ) were calculated. The LOD and LOQ were, respectively, 0.6 and 1.8 μg/L for Pb; 0.02 and 0.06 μg/L for Cd; and 0.05 and 0.15 mg/L for Zn. 

### 2.3. PAH Determination

To determine the PAH content in the honeys, the method previously used by Dorina et al. [63] was employed. The content of total PAHs was determined as the sum of the concentrations of the compounds benzo(a)anthracene, chrysene, benzo(b)fluoranthene and benzo(a)pyrene, using high-performance liquid chromatography with fluorescence detection (HPLC-FLD). First, 10 g of honey was extracted using 25 mL hexane (for HPLC, purity ≥ 99.9, Sigma-Aldrich, Germany) in an ultrasonic bath for 60 min. The supernatant was purified on a Florisil (Merck, Germany) clown and then evaporated to dry in a stream of nitrogen. The sample was reconstituted using 1 mL of acetonitrile. Before being injected, the sample was filtered using a 0.45 µm filtration cartridge. A Perkin Elmer 200 Series High-Performance Liquid Chromatograph (HPLC) with an FLD detector was used (Perkin-Elmer, Shelton, CT, USA). The determination conditions were as follows: flow rate, 1.4 mL/min; gradient mobile phase of H_2_O and acetonitrile; column temp., 24 °C; injection volume, 50 µL; column, INERTSIL ODS-P 5 um 15 cm × 0.46 cm, TEKNOKROMA. Different wavelengths appropriate for each compound for the FLD detector were used. PAH calibration mix CRM47940 (Supelco, Germany) was used. The lower limit of the measurement range for this method (MDL), together with the expanded uncertainty, was 10 ± 0.03 µg/kg for benzo(a)anthracene, 10 ± 0.04 µg/kg for chrysene, 10 ± 0.04 µg/kg for benzo(b)fluoranthene, 10 ± 0.02 µg/kg for benzo(a)pyrene and 10 ± 0.04 µg/kg for the sum of PAHs.

### 2.4. Statistical Analysis

All chemical analyses were performed in triplicate. From the obtained results, the mean and standard deviation were calculated using the Statistica v. 13.3 software (StatSoft, Tulsa, OK, USA). A one-way analysis of variance (ANOVA) was conducted to investigate the effect of the apiary location on the heavy metal content. The significance of differences between the means was estimated by the Tukey test (*p* ˂ 0.05).

## 3. Results

In Table 1, the average content of particular toxic metals is shown. The zinc content in the honeys tested ranged from about 2 to 4.5 mg/kg. Currently, the zinc content in honey is not regulated; the previously applicable Polish Regulation of the Minister of Health [64] allowed up to 20 mg/kg of honey. In light of these requirements, the determined content can be considered low. The average Pb content in the tested honeys was highly diversified and ranged from 0.0035 mg/kg (3T honey) to 0.388 mg/kg (6W honey). Lead is the only toxic metal whose content in honey is regulated by Commission Regulation (EU) 2023/915 regarding the maximum permitted levels of certain contaminants in food [60]. The lead content was shown to exceed the permitted content in 11 of the 35 honeys tested, and the maximum concentration, shown in the 6W honey sample, exceeded the permitted limit by more than three times.

The cadmium content in the honey samples tested also showed wide variation and ranged from approximately 0.0005 to 0.064 mg/kg. Similarly to the zinc content, the cadmium content is not currently regulated; however, legislation in the EU [65] that is no longer valid allowed up to 0.03 mg of cadmium/kg of honey. This value was exceeded in two samples obtained from an apiary located in the Silesia Voivodeship, and the maximum concentration, found in sample 2A (0.06453 mg/kg), was therefore twice as high as this limit.

**Figure 2:**
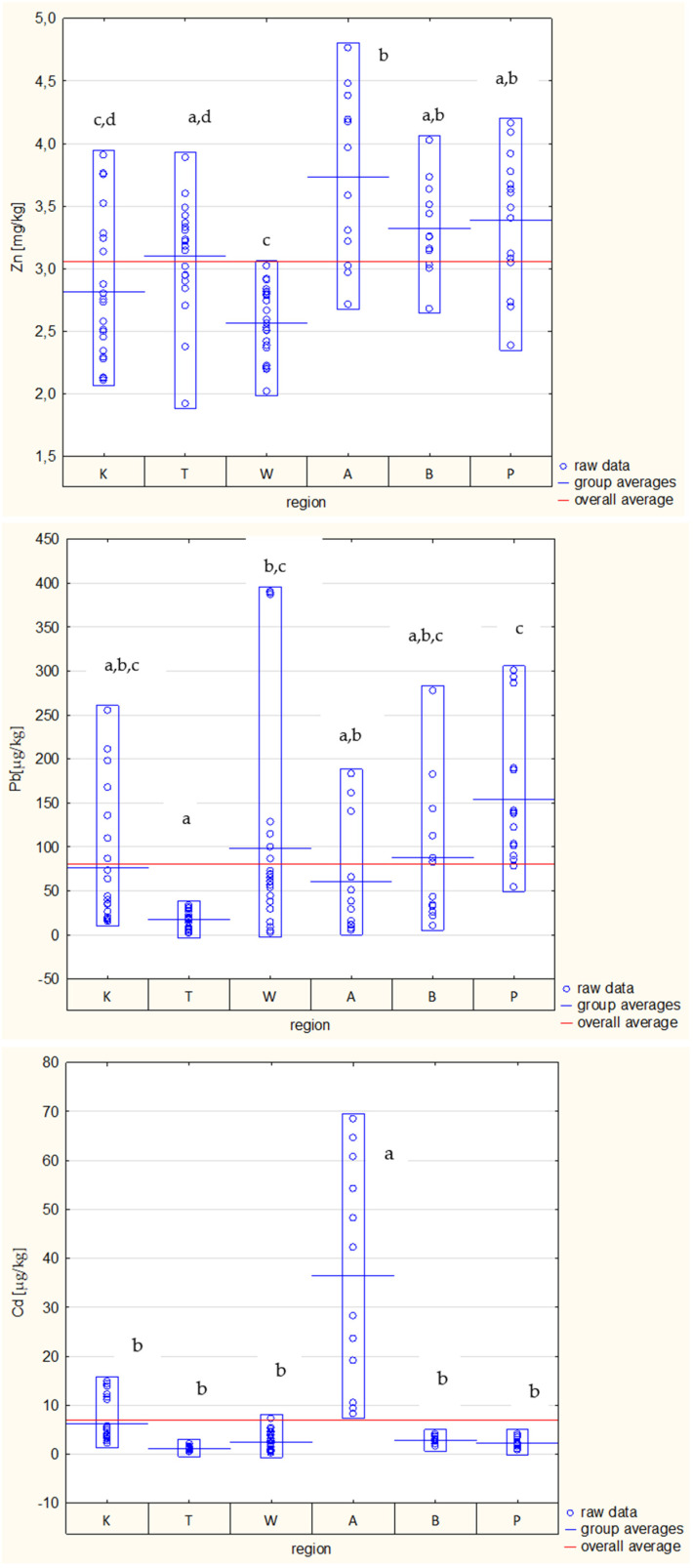
Content of zinc (Zn), lead (Pb) and cadmium (Cd) in honeys from different regions (K—Cracow, T—Tri-City, W—Warsaw, A—Katowice, B—Szczytno, P—Poznań); different letters (a, b, c, d) indicate homogeneous groups, separated as a result of *post hoc* analysis (Tukey test).

As can be seen, the content of individual metals in particular samples showed wide variation, and the zinc content was the least varied. Based on the results of the one-way ANOVA, significant differences (*p* < 0.05) were observed among the concentrations of trace metals in samples coming from different locations (Figure 2), although, only in the case of cadmium contamination, can it be clearly stated that its content was the highest in samples from the Silesian Voivodeship.

In the collected samples, no detectable levels of the four PAHs studied (benzo(a)anthracene, chrysene, benzo(b)fluoranthene, benzo(a)pyrene) were found. PAHs are products of the incomplete combustion and pyrolysis of organic matter, originating from anthropogenic and natural sources. The presence of PAHs in the environment has attracted much attention in recent decades due to their persistence and toxic, mutagenic and carcinogenic properties. Honey may be contaminated with such substances as a result of environmental pollution (forest fires, stubble burning, location of hives near industrial areas, etc.) or beekeeping practices, such as blowing smoke into hives during service to calm the bees. Contrary to our expectations, the results obtained indicate that honeys from urbanized areas do not contain these harmful substances.

## 4. Discussion

It can be stated that all collected honey samples contained specific amounts of harmful metals. Cadmium (Cd) and lead are among the most toxic elements in honey that have an anthropogenic origin. Among the studied metals, zinc is the only one that is essential in some biochemical transformations, and it is necessary for the proper functioning of the human body; only in excessive amounts can it be harmful to health. The detected zinc concentration (2–4.5 mg/kg) was comparable to those reported by other researchers [66,67,68,69,70]. Bosancic et al. [66] stated that the content of zinc (Zn) was significantly higher in analyzed honeys taken near a thermal power plant (1.36 mg/kg) compared with other analyzed honeys. They showed that the zinc (Zn) content in honeys from organic apiaries was much lower and ranged around 0.5 mg/kg. In honeys from Ethiopia, Tibebe et al. [70] detected zinc (Zn) in concentrations of about 2 mg/kg, and Beshow et al. [67] found concentrations of about 1.4–4 mg/kg. It can therefore be concluded that the zinc (Zn) concentration that we detected is typical for honeys from industrialized areas. However, as we note in the Results section, it is much lower than the standards that were previously in force.

Among the metals determined, the lead (Pb) content most often exceeds the limit specified in legal standards, which is currently 0.1 mg/kg, and only honeys from the Tri-City and Katowice contained, on average, less lead (Pb) than the permissible limit (Figure 2). However, these results are not surprising. According to Šerevičiene et al. [15], the Pb content in honey from urbanized areas can reach up to 1.650 mg/kg. In our own research, conducted in 2003–2004 in several dozen samples of bee honeys from all over Poland, it was shown that the lead content in honey often exceeds the permissible limits. The most lead was found in honeys from industrialized areas [71]. Tomczyk et al. [72] found that the lead (Pb) content in honeys from the Podkarpackie Voivodeship (Southeast Poland) varied from 0.00 to 0.77 mg/kg; they found also that the metal content in honeys that originated from ecological and urbanized areas were comparable. According to Purcarea et al. [73], the Pb level in Polish honey ranges from 0.002 to 0.098 mg/kg, and that in Romanian honey ranges from 0.018 to 0.05 mg/kg, which is lower than in the honeys that we tested. However, they stated that the geographical origin and soil composition strongly influence honey’s chemical composition, and the crucial factor for heavy metal transfer seems to be the soil pH.

The main source of lead (Pb) in the environment is heavy industry (mining, metallurgy, chemical industry, tanning) and car exhaust fumes. Within industrial centers and communication routes, the soil and plant contamination is 300 times greater than elsewhere, which is why the content of this metal may be increased in honeys from apiaries located in urbanized areas, especially near communication routes. All of the tested honeys came from apiaries located in city centers or near major transport routes, which explains the high lead (Pb) contamination.

The determined cadmium (Cd) concentration showed large fluctuations and ranged between 0.0005 and 0.065 mg/kg. An excess of cadmium content was noted in two samples (2A, 4A) coming from Katowice, which is located in the center of the Upper Silesian Industrial Region. The average cadmium content in samples from this region was also significantly higher than that from other regions. Cadmium (Cd) is one of the toxic metals for which a biological necessity has not yet been demonstrated. Its main sources in the environment are the mining and metallurgical industries. The Upper Silesian Industrial Region is the largest industrial region in Poland. It includes industrial units, mainly mines and steelworks in the central–eastern part of the Silesian Voivodeship. The concentrations of cadmium (Cd) in honey samples from the Silesia region were consistent with the values obtained in the Podkarpackie region (Southeast Poland), which ranged from 0.01 to 0.07 mg/kg [72]. The cadmium (Cd) content in honey samples collected in other regions does not differ significantly from that reported by other researchers. Kováčik et al. [74] found 0.0524 mg Cd/kg in forest honey from the vicinity of an industrial town. In the research of Tesauro et al. [24], the range of concentrations for cadmium (Cd) was 0.006–0.040 mg/kg; according to Purcarea et al. [73], it was even slightly lower (0.007–0.021 mg/kg). Nowak et al. [75] stated that the cadmium (Cd) content in honeys from Lower Silesia (Southwest Poland) did not exceed the permissible values (0.03 mg/kg). Moreover, according to Ligor et al. [76], the cadmium (Cd) concentration was at a level below the background equivalent concentration.

In the current study, information about honey’s contamination with potentially toxic elements has been presented. Tomczyk et al. [72] claim that the bodies of bees create an effective barrier to the migration of heavy metals from the environment to honey; because of this, the honey is free from pollution and safe for human consumption. However, we have once again confirmed that honeys from urbanized areas may contain larger amounts of harmful substances, especially cadmium (Cd) and lead (Pb), than honeys from other areas. It could be seen, therefore, that the locations of apiaries determine the nutritional and health properties of honey. On the other hand, taking into account the very low consumption of honey (estimated in Poland at approximately 400 g/person/year), it can be concluded that there is no risk of exceeding the toxicological values. The most contaminated honeys from urban areas contain 0.388 mg/kg cadmium (Pb) and 0.065 mg/kg cadmium (Cd); the weekly intake of these metals from honey is 0.00030 and 0.00006 mg/kg body mass, respectively, which is about 0.2 and 21.2% of the PTWI, respectively (Table 2).

PAHs occur in all parts of the environment, and their presence is also detected in many food products, mainly of animal origin [55,56,57]. The PAH content in honey has been determined only a few times so far [23,52,59,63,77,78,79]. Similarly to the research of Corredera et al. [23], our research has shown that the PAH content in honey is below the detection level. Moreover, Dorina et al. [63] did not detect benzo[a]pyrene, the representative marker of total PAHs, in their examined honey samples. Ciemniak et al. [78], examining honeys from West Pomerania (Northwest Poland), showed that honey contained mostly non-carcinogenic PAHs of low molecular weight. According to Batelková et al. [79], the concentrations of individual PAHs in honey samples coming from the Czech Republic ranged between 0.02 and 1.93 μg/kg. Based on our results and the abovementioned research, it can be stated that honey is not a product that accumulates these health-threatening contaminants. This may be due to the fact that PAHs are highly soluble in fats and therefore honey does not accumulate them.

## 5. Conclusions

Honey is a natural food product with many health-promoting properties. Despite its many benefits, honey can be contaminated with chemical compounds, including harmful metals and organochlorine compounds, which is the result of soil and plant contamination. Our studies took into account, in particular, the contamination of honey with heavy metals (lead (Pb), cadmium (Cd) and zinc (Zn)) and polycyclic aromatic hydrocarbons (PAHs). Based on the conducted tests, it was found that honeys coming from urbanized areas contained certain amounts of toxic metals (lead (Pb) and cadmium (Cd)); however, taking into account the very low consumption of honey, they do not pose a risk to human health. None of the tested honeys contained PAHs above the detection level; thus, consumers should not be concerned about PAHs in honey. However, even though the content of the toxic metals was not high and therefore may not pose a risk to human health, attention should be paid to the locations of apiaries.

## Figures and Tables

**Table 1:** Average content of zinc (Zn), cadmium (Cd) and lead (Pb) in tested honey samples.

Sample No.	Zn [mg/kg]	Pb [mg/kg]	Cd [mg/kg]
1T	3.36	0.00743	0.00117
2T	3.33	0.01974	0.00091
3T	2.95	0.00353	0.00169
4T	2.71	0.01851	0.00056
5T	2.90	0.03012	0.00118
6T	3.31	0.02686	0.00131
7T	3.18	0.01633	0.00148
1W	2.92	0.00856	0.00073
2W	2.53	0.08636	0.00215
3W	2.50	0.06476	0.00222
4W	2.19	0.11450	0.00460
5W	2.79	0.03754	0.00097
6W	2.51	0.38895	0.00049
7W	2.56	0.05673	0.00407
8W	2.51	0.02916	0.00515
1A	2.97	0.03853	0.00938
2A	4.17	0.16181	0.06463
3A	3.30	0.01078	0.02370
4A	4.48	0.02928	0.04831
1K	3.52	0.01897	0.00229
2K	2.34	0.01687	0.01165
3K	2.51	0.02632	0.00537
4K	2.50	0.08664	0.00313
5K	3.24	0.13555	0.00328
6K	2.29	0.03982	0.00401
7K	3.28	0.21158	0.01438
1P	3.63	0.13901	0.00378
2P	3.05	0.07793	0.00217
3P	2.73	0.13747	0.00198
4P	3.92	0.12239	0.00157
5P	3.60	0.29339	0.00164
1B	3.16	0.18249	0.00164
2B	3.14	0.11282	0.00357
3B	3.73	0.03414	0.00293
4B	3.26	0.02160	0.00318
Requirement *	-	≤0.100	-

Source: own research; regions K—Cracow, T—Tri-City, W—Warsaw, A—Katowice, B—Szczytno, P—Poznań; red color indicates values exceeding the permissible limits; * [57].

**Table 2:** Estimation of selected heavy metal intake with honey from urbanized areas.

Metal	Concentration [mg/kg]		Weekly Intake [mg/kg b.m.] *	PTWI	% PTWI
Pb	mean	0.080	1.02 × 10^−5^	0.025	0.04
	max	0.389	4.97 × 10^−5^	0.025	0.2
Cd	mean	0.007	8.95 × 10^−7^	0.007	0.1
	max	0.065	8.30 × 10^−6^	0.007	1.2
Zn	mean	3.06	3.09 × 10^−4^	1	0.03
	max	4.48	5.73 × 10^−4^	1	0.06

Source: own evaluation; PTWI—provisional tolerance weekly intake (acc. WHO); * weekly intake was evaluated for an adult with an average body weight of 70 kg.

## Data Availability

The original contributions presented in this study are included in the article. Further inquiries can be directed to the corresponding author.
